# Inhibition of STAT3 augments antitumor efficacy of anti-CTLA-4 treatment against prostate cancer

**DOI:** 10.1007/s00262-021-02915-6

**Published:** 2021-03-31

**Authors:** Kristina Witt, Susan Evans-Axelsson, Andreas Lundqvist, Martin Johansson, Anders Bjartell, Rebecka Hellsten

**Affiliations:** 1 Department of Oncology-Pathology, Karolinska Institutet, Stockholm, Sweden; 2 Department of Medicine, Huddinge, Center for Hematology and Regenerative Medicine, Karolinska Institutet, Stockholm, Sweden; 3 Division of Urological Cancers, Institution of Translational Medicine, Lund University, Malmö, Sweden; 4 Aqilion AB, Helsingborg, Sweden

**Keywords:** STAT3, Immunotherapy, Prostate cancer, Anti-CTLA-4, Treg, STAT3 inhibitors, Small molecule inhibitor

## Abstract

There is an urgent need for new treatment options in metastatic drug-resistant prostate cancer. Combining immunotherapy with other targeted therapies may be an effective strategy for advanced prostate cancer. In the present study, we sought to investigate to enhance the efficacy of anti-CTLA-4 therapy against prostate cancer by the combination with STAT3 inhibition.

Male C57BL6 mice were subcutaneously inoculated with the murine prostate cancer cell line RM-1. Tumor progression was monitored following treatment with vehicle, the small molecule STAT3 inhibitor GPB730, anti-CTLA-4 or GPB730 + anti-CTLA-4. Treatment with anti-CTLA-4 or anti-CTLA-4 + GPB730 significantly inhibited tumor growth and enhanced survival compared to vehicle. Combining anti-CTLA-4 treatment with GPB730 resulted in a significantly prolonged survival compared to anti-CTLA-4 alone. GPB730 significantly increased infiltration of CD45 + cells in tumors of anti-CTLA-4-treated mice compared to anti-CTLA-4 alone. The levels of tumor-infiltrating Tregs were significantly decreased and the CD8:Treg ratio significantly increased by GPB730 treatment in combination with anti-CTLA-4 compared to anti-CTLA-4 alone. Immunohistochemical analysis showed a significant increase in CD45-positive cells in anti-CTLA-4 and anti-CTLA-4 + GPB730-treated tumors compared to vehicle or GPB730 monotherapy. Plasma levels of IL10 were significantly increased by anti-CTLA-4 compared to vehicle but no increase was observed when combining anti-CTLA-4 with GPB730.

In conclusion, STAT3 inhibition by GPB730 enhances the antitumoral activity of anti-CTLA-4 and decreases the intratumoral Treg frequency in a prostate cancer mouse model. These results support the combination of STAT3 inhibition with anti-CTLA-4 therapy to increase clinical responses in patients with prostate cancer.

**Supplementary Information:**

The online version contains supplementary material available at 10.1007/s00262-021-02915-6.

## Introduction

We need novel and effective treatment options when metastatic prostate cancer becomes resistant to androgen deprivation therapy (ADT) and to chemotherapy. Combining immunotherapy with other targeted therapies may be an effective strategy for advanced prostate cancer.

Immune checkpoint inhibitors e. g. anti-CTLA-4 and anti-PD-1 are effective therapeutics in several cancers [1]. These antibodies block inhibitory signals on cytotoxic T-cells and thus enhance the immune response toward cancer cells. However, checkpoint inhibitors as monotherapy have not yet been proven to be of substantial clinical benefit in patients with prostate cancer [2–5], spurring investigations into new strategies to modulate the immunological response. Combinations of established anti-cancer treatments and check-point inhibitors have been suggested for improved immunotherapeutic effect [6]. The anti-PD-1 antibody pembrolizumab in combination with anti-androgen therapy has shown some efficacy in a subset of metastatic prostate cancer patients [7]. Attempts have been made with the anti-CTLA-4 antibody ipilimumab plus radiation therapy, ADT and other checkpoint inhibitors (anti-PD-1), although with limited enhanced treatment effects in prostate cancer [8]. However, a follow-up study with ipilimumab in combination with radiation therapy showed prolonged survival in a portion of patients compared with radiation therapy alone [9]. Furthermore, a recent report shows that ipilimumab in combination with the anti-PD-1 antibody nivolumab has antitumoral activity in a subset of patients with metastatic prostate cancer [10].

The STAT3 signaling pathway is involved in the induction and function of immunosuppressive cells and the inhibition of dendritic cell functions in the tumor microenvironment, thus promoting immune evasion in cancer [11]. Furthermore, STAT3 regulates immunosuppressive factors from the tumor cells themselves [12, 13]. STAT3 has been suggested as a promising drug target for several types of cancer [13, 14]. By modulating both immunosuppressive cells and the immunosuppressive function of cancer cells, inhibition of STAT3 may also potentiate the effect of immune checkpoint inhibitors.

We have demonstrated that the small molecule STAT3 inhibitor galiellalactone [15] inhibits the prostate cancer cell induced generation of monocytes with a myeloid-derived suppressor cell (MDSC)-like phenotype and immunosuppressive factors in both human prostate cancer cells and immune cells ex vivo [16]. Recently, the semisynthetic galiellalactone analogue GPB730 has been shown to inhibit the immunosuppressive activity of regulatory NK cells and decrease pSTAT3-S727 expression in these cells ex vivo [17]. These results demonstrate the promise of small molecule STAT3 inhibitors to reduce immunosuppression in the microenvironment, thus potentially enhancing the antitumoral response to immunotherapies.

In the present study, we investigated the possibility of enhancing the efficacy of anti-CTLA-4 therapy in a syngeneic prostate cancer mouse model by combining treatment with the STAT3 inhibitor GPB730.

## Materials and methods

### Cell culture

The murine prostate cancer cell line RM-1 (American Type Culture Collection, ATCC CRL-3310) was used. Cells were maintained in Dulbecco's Modified Eagle's Medium (Cytiva, Hyclone Laboratories, South Logan, UT, USA) supplemented with 10% fetal bovine serum (Biological Industries, Israel) and 1% penicillin–streptomycin (Cytiva, Hyclone Laboratories, South Logan, UT, USA) and were grown at 37 °C in a humidified atmosphere with 95% O_2_ and 5% CO_2_. Cells were routinely tested and found free of mycoplasma.

#### In vivo syngeneic mouse model

Male C57BL6 mice (Janvier laboratories, Le Genest-Saint-Isle, France) were subcutaneously inoculated in the flank with 100 000 RM-1 cells in a mixture of Matrigel (Corning, Thermo Fisher Scientific, Waltham, MA, USA) and cell culture medium in the ratio 1:1. On day four post inoculation, mice were randomized and treated with either vehicle (2% DMSO) in phosphate buffered saline (PBS), 5 mg/kg GPB730 daily intraperitoneal (ip) injections, 10 mg/kg anti-CTLA-4 (clone 9H9; Bio X Cell, Lebanon, NH, USA) ip on day 4, 7, 10 and 13 post tumor cell inoculation or the combination of GPB730 and anti-CTLA-4. The survival study comprised of 11 mice per treatment group. Tumors were measured 2–3 times per week using a caliper. Mice were sacrificed if tumor volume exceeded 1000 mm^3^ by caliper measurement or the appearance of tumor ulcerations. For survival study, the endpoint was defined as a tumor size of 1000 mm^3^ or tumor ulcerations. For the immune profile and immunohistochemical studies, mice were sacrificed after 2 weeks of treatment. The immune profile studies comprised of 6 mice per treatment group and the immunohistochemical study of 5–9 mice per treatment group. Mice were kept on a 12 h light − dark cycle with access to food and water ad libitum. Experimental procedures were approved by the Regional Ethics Committee for Animal Research at Lund University, Sweden (permit number M134-14). The RM-1 prostate cancer model was used throughout these studies as it represents a suitable model to study androgen-independent aggressive prostate cancer and immuno-oncology.

GPB730 was provided by Glactone Pharma Development AB (Gothenburg, Sweden). GPB730 is a semisynthetic analogue of galiellalactone, a direct small molecule inhibitor of STAT3 [15]. In contrast to most other STAT3 inhibitors (e. g. Stattic and AG490) which inhibit phosphorylation and upstream activation factors of STAT3, GPB730 does not affect phosphorylation of STAT3 but rather exerts its inhibitory actions by binding to STAT3 thus blocking binding to DNA and preventing transcription of STAT3 regulated genes. Molecular structure of GPB730 is presented in Neo et al. [17].

### Immunohistochemical and immunofluorescence analysis

Formalin fixed and paraffin embedded RM-1 mouse tumors were subjected to immunohistochemistry (IHC) using Dako Autostainer Plus En VisionTM + Kit (Dako, Glostrup, Denmark) and stained with the antibodies anti-pSTAT3-T705 (ab76315 Abcam, Cambridge, UK), anti-pSTAT3-S727 (#9143 Cell Signaling Technology, Danvers, MA, USA), anti-FOXP3 (ab54501 Abcam) and anti-CD45 (ab25386 Abcam). The immunostainings were analyzed using Halo image analysis software (Indica Laboratories, Albuquerque, NM, USA).

For immunofluorescent staining, the RM-1 tumors were stained using the Opal Multiplex immunofluorescence assay (Akoya Biosciences) and imaged with Mantra multispectral image system (Akoya Biosciences). Antibodies used were anti-CD3E (LS-C343957 LSBio), anti-CTLA-4 (ab 237,712 Abcam) and anti-pSTAT3-S727 (#9143 Cell Signaling Technology).

### Flow cytometry analysis of mouse tumor tissue and spleen

Single cell suspensions were prepared from mouse tumors and spleens. Briefly, tumors were dissociated using a buffer consisting of 2 mg/ml Dispase II (Gibco, Thermo Fisher Scientific, Waltham, MA, USA), 100 μg/ml DNase I (Sigma-Aldrich, Merck, Darmstadt, Germany) and 0.2 mg/ml Collagenase (Gibco). The cell suspension was filtered through a 70 μm filter before washed and incubated with Red Blood Cell lysis (Gibco), washed in FACS buffer (2–5% FBS in PBS) before proceeding to the staining for flow cytometry analysis. Spleens were passed through a 70 μm strainer and incubated in BD Pharm Lysing Solution (BD Biosciences, Franklin Lakes, NJ, USA) before washed in PBS and resuspended in FACS buffer. Cells were incubated with mouse Fc block (anti-CD16/32; BD Biosciences) before extracellular staining. For intracellular staining, the cells were fixed and permeabilized according to the manufacturer’s protocol (Invitrogen). Single cell suspensions of tumor and splenocytes were stained according to standard flow cytometry protocol. Antibodies used for flow cytometry analyses are listed in supplementary table 1. Dead cells were excluded using the LIVE/DEAD fixable Aqua Dead Cell Stain (Thermo Fisher Scientific, Waltham, MA, USA). Flow cytometry analysis of dissociated mouse tumors and spleens was performed on FACSVerse (BD Biosciences) or LSRII (BD Biosciences). Samples were blinded prior acquisition and kept blinded during analysis. Data were analyzed with FlowJo™ Software v10.0 (Becton, Dickinson and Company, Ashland OR, USA). Gates were set based on unstained and dead cell marker only stained controls in both spleen and tumor samples. Gating strategies for the different immune cell populations are shown in supplementary Fig. 3.

### Cytometric bead array and ELISA for inflammatory cytokines and chemokines in mouse plasma

Plasma samples were prepared on the day of sacrifice. Blood was collected at time of sacrifice and transferred to tubes with 10% EDTA (0.5 M). Samples were centrifuged for 15 min at 1500 × g at 4 °C. Plasma was aliquoted and stored at −80 °C. Cytokine levels were evaluated using Cytometric Bead Array Mouse Inflammatory Cytokines according to the manufacturer’s instructions (BD Biosciences, Franklin Lakes, NJ, USA). The cytokines IL6, tumor necrosis factor (TNF), IL10, IL12p70, monocyte chemoattractant protein 1 (MCP-1) and IFNγ were analyzed. The levels of cytokines were evaluated using FACSVerse (BD Biosciences) or CytoFLEX (Beckman Coulter, Brea, CA, USA) with subsequent analysis using FlowJo v10.0 software. Tumor growth factor *ß* (TGFß) and CXCL10 in mouse plasma was analyzed by ELISA (Abcam, Cambridge, UK) according to the manufacturer’s protocol. Plasma from 5 non-treated mice without tumors and 8–10 tumor bearing mice per treatment group were analyzed.

### Statistics

Statistical analysis was performed using GraphPad Prism and ANOVA with Dunnett´s multiple comparison test. Data are presented as mean ± standard error of the mean (SEM). Correlations analysis were performed using Pearson correlation analysis. Analysis of the survival study was performed using log rank Mantel Cox test. Statistical significance was considered when *p* ≤ 0.05.

## Results

### GPB730 enhances the antitumoral effect and increases survival in anti-CTLA-4-treated mice

**Fig. 1:**
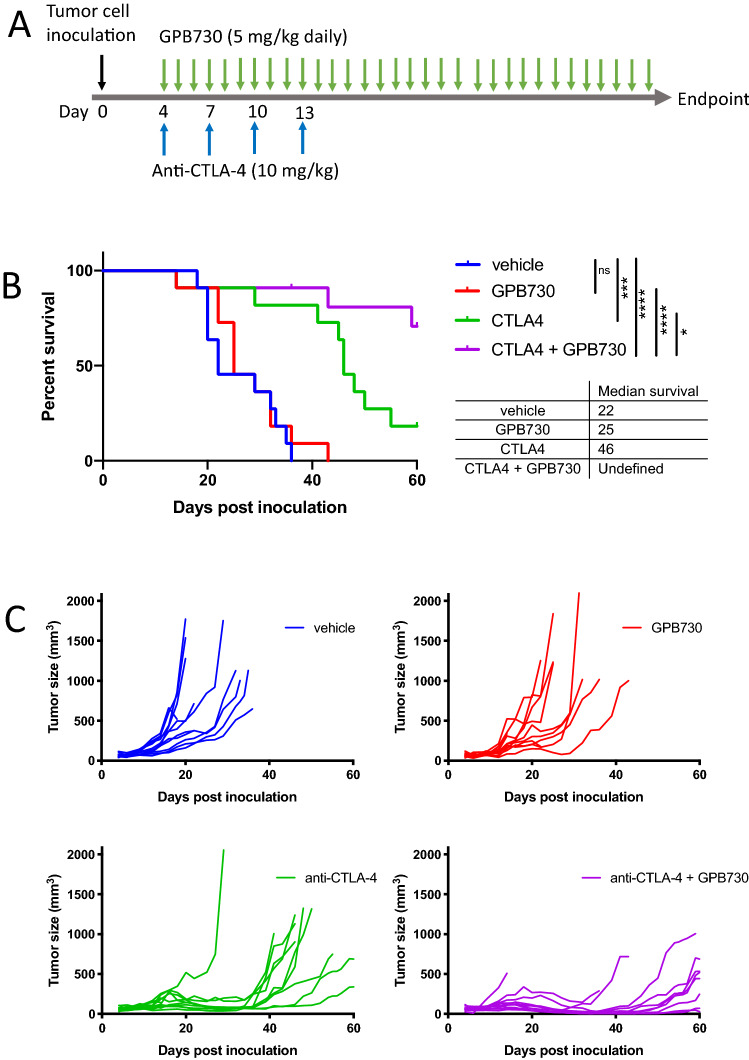
GPB730 increases survival and enhances the antitumoral response to anti-CTLA-4. Mice inoculated with RM-1 were treated with vehicle, GPB730, anti-CTLA-4 or anti-CTLA-4 + GPB730 with treatment start 4 days post inoculation. **a** In vivo treatment schedule. **b** Survival of mice with RM-1 tumors in indicated treatment groups (vehicle *n* = 11; GPB730 *n* = 11; anti-CTLA-4 *n* = 11, anti-CTLA-4 + GPB730 *n* = 11). Statistical analysis of survival was performed using Log rank Mantel-Cox test (ns *p* > 0.05; *p ≤ 0.05; ***p ≤ 0.0001; ****p ≤ 0.0001). Tick mark indicates censored event. **c** Individual RM-1 tumor growth per treatment group. Each line represents individual tumor growth

We investigated the effect of combining anti-CTLA-4 treatment with the STAT3 inhibitor GPB730 on tumor growth in a prostate cancer mouse tumor model (Fig. 1). C57BL6 male mice with subcutaneous RM-1 tumors were treated with vehicle, GPB730, anti-CTLA-4 or GPB730 + anti-CTLA-4 according to the treatment schedule in Fig. 1a. Mice treated with anti-CTLA-4 showed significantly increased survival time (median 46 days) compared to vehicle treated mice (median 22 days) (Fig. 1b). Two mice in the combination treatment group showed tumor regression (Fig. 1c). When combining anti-CTLA-4 with the STAT3 inhibitor GPB730 a significantly increased survival time was observed compared to anti-CTLA-4 treatment alone. GPB730 treatment alone had no significant effect on survival (median 25 days) compared to vehicle (Fig. 1b). The effect of combination treatment on long-term survival could not be evaluated due to the ethical guidelines. There was no weight loss in mice in any group during the survival study (supplementary Fig. 1a).

### IHC analysis of mouse tumors for pSTAT3, FOXP3 and CD45 expression

**Fig. 2:**
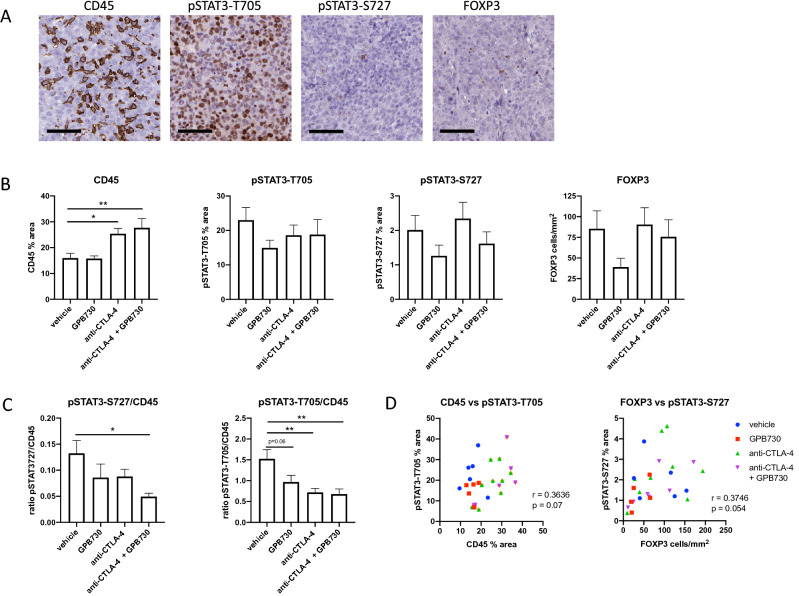
CD45, pSTAT3 and FOXP3 expression in RM-1 tumors. IHC staining of RM-1 tumors from mice treated with vehicle, GPB730, anti-CTLA-4 or anti-CTLA-4 + GPB730 for 2 weeks **a** Representative images of CD45, pSTAT3-T705, pSTAT3-S727 and FOXP3 IHC immunostainings of RM-1 tumors. Scale bar indicates 70 µm. **b** IHC quantitative analysis of CD45, pSTAT3-T705, pSTAT3-S727 and FOXP3 immunostaining in tumors from mice treated with vehicle, GPB730, anti-CTLA-4 or anti-CTLA-4 + GPB730 for 2 weeks. Images quantified using HALO image analysis. Data presented as mean ± SEM. Vehicle *n* = 6; GPB730 *n* = 5; anti-CTLA-4 *n* = 9; anti-CTLA-4 + GPB730 *n* = 6–7. One-way ordinary ANOVA with Dunnetts’s multiple comparisons test in comparison with vehicle group (*p ≤ 0.05; **p ≤ 0.001). C. Ratio of pSTAT3 and CD45 expressing cells in tumors. Data presented as mean ± SEM. Vehicle *n* = 6; GPB730 *n* = 5; anti-CTLA-4 *n* = 9; anti-CTLA-4 + GPB730 *n* = 6. One-way ordinary ANOVA with Dunnetts’s multiple comparisons test in comparison with vehicle group (*p ≤ 0.05; **p ≤ 0.001). D. Correlations between CD45 and pSTAT3-705 and FOXP3 and pSTAT3-S727 immunostainings in RM-1 tumors using Pearson correlation analysis

To uncover any potential underlying mechanisms of the improved overall survival in mice treated with anti-CTLA-4 + GPB730, the expression of CD45, pSTAT3-T705, pSTAT3-S727 and FOXP3 in RM-1 tumors of mice treated for 2 weeks with vehicle, GPB730, anti-CTLA-4 or anti-CTLA-4 + GPB730 was analyzed by IHC analysis (Fig. 2). The tumors were densely infiltrated with CD45-positive cells, and the observed CD45-positive area ranged from 10 to 37% of the tumor area (Fig. 2a, b). The CD45 stained area was significantly larger in both anti-CTLA-4 and anti-CTLA-4 + GPB730-treated tumors compared to vehicle, with the greatest infiltration of CD45-positive cells in the combination treatment group. The differences in CD45 expression between anti-CTLA-4 and anti-CTLA-4 + GPB730 were not significant.

pSTAT3-T705 was found in the nucleus and comprised 6% to 40% of the tumor area (Fig. 2a, b). No evident overlap of areas with cells positive for pSTAT3-T705 and CD45-positive areas was observed (supplementary Fig 2a). The ratio pSTAT3-T705 to CD45 cells was significantly reduced by anti-CTLA-4, combination treatment of anti-CTLA-4 + GPB730, and numerically reduced by GPB730 alone (Fig. 2c). There was no correlation between CD45 expression and STAT3 phosphorylation at T705 expression (Fig. 2d).

Cells stained positive for pSTAT3-S727 were scattered within the tumors and expression was mainly cytoplasmic although strong nuclear staining was occasionally observed (Fig. 2a, supplementary Fig. 2b). The total area of pSTAT3-S727-positive cells did not significantly differ between treatments groups (Fig. 2b). However, the ratio of pSTAT3-S727 to CD45 was significantly decreased in the GPB730 + anti-CTLA-4 treatment group compared to vehicle, but not in the other treatment groups (Fig. 2c).

Strong nuclear FOXP3 staining, representing Tregs, was observed in cells scattered throughout the tumors (Fig. 2a). The density of FOXP3 expressing cells in tumors did not significantly differ between treatment groups, although a trend toward a decrease in density was observed by GPB730 (Fig. 2b). A weak correlation between pSTAT3-S727 and FOXP3 expression was observed in mouse tumors (Fig. 2d).

**Fig. 3:**
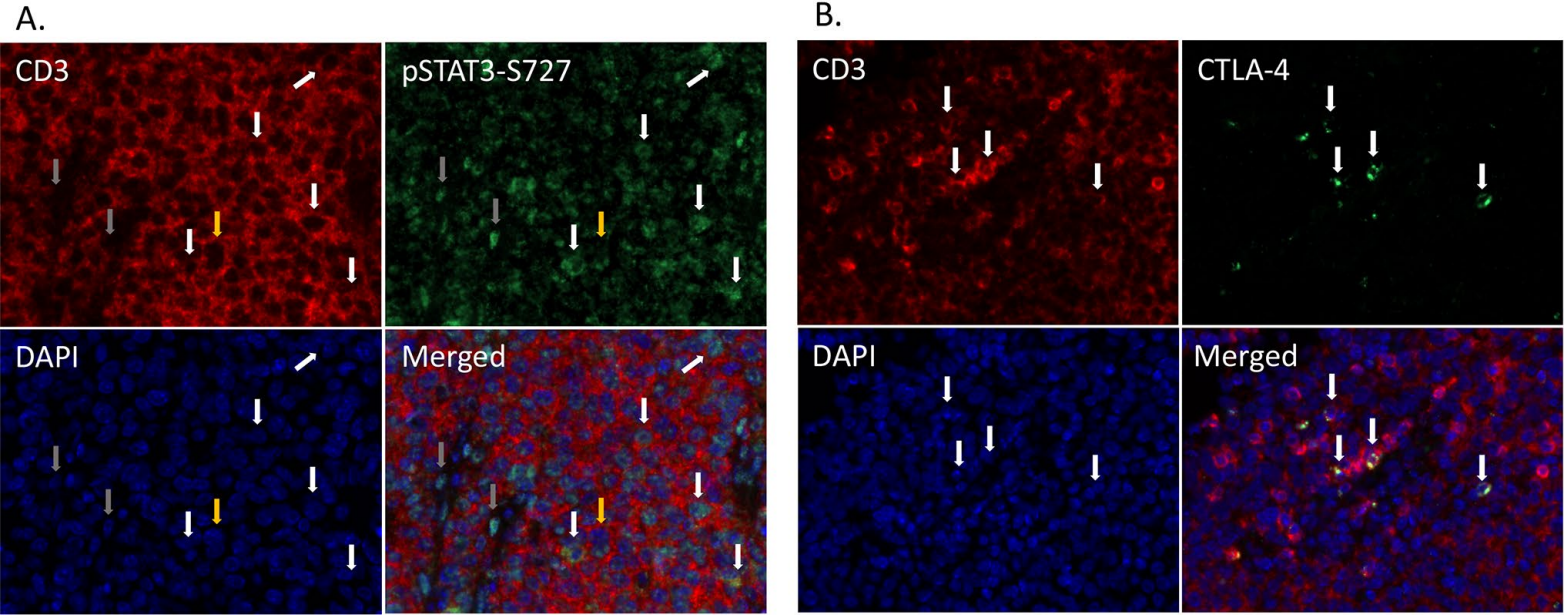
Expression pSTAT3-S727 and CTLA-4 in CD3 cells. **a** Immunofluorescent double staining for CD3 and pSTAT3-S727 in RM-1 tumors. Images show CD3 (red), pSTAT3-S727 (green), nuclear staining with DAPI (blue) and merged image. The white arrows indicate cells stained for both CD3 and pSTAT3-S727, grey arrows indicate pSTAT3-S727 expressing cells negative for CD3 and yellow arrows indicate CD3-positive cells with no pSTAT3-S727 expression. **b** Immunofluorescent double staining for CD3 and CTLA-4 in RM-1 tumors. Images show CD3 (red), CTLA-4 (green), nuclear staining with DAPI (blue) and merged image. The arrows indicate cells stained for both CD3 and CTLA-4. Images are taken at 40X magnification (colour figure online)

pSTAT3-S727 was observed to be expressed in a portion of CD3-positive cells in the tumor microenvironment as well as in a few scattered CD3 negative cells as detected by immunofluorescence (Fig. 3a). A portion of CD3-positive cells in RM-1 tumors were also observed to express CTLA-4 (Fig. 3b).

### GPB730 increases infiltration of CD45 + cells in tumors of anti-CTLA-4-treated mice

**Fig. 4:**
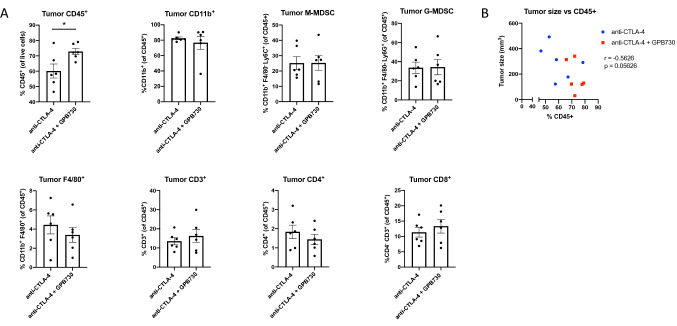
GPB730 increases infiltration of CD45 + cells in tumors of anti-CTLA-4-treated mice. Flow cytometry analysis of the immune cell composition in RM-1 tumors of mice treated with anti-CTLA-4 or anti-CTLA-4 + GPB730 for 2 weeks with treatment start 4 days post inoculation. **a** Infiltration of CD45 + cells in tumors and the frequency of different immune cell populations among CD45 + gated cells in tumors (anti-CTLA-4 *n* = 6; anti-CTLA-4 + GPB730 *n* = 6). Data presented as mean ± SEM. **b** Correlation of CD45 + with tumor size using Pearson correlation analysis (*p ≤ 0.05)

To gain further understanding of the immune cell profile, RM-1 tumors and spleens from mice treated with anti-CTLA-4 alone or in combination with the STAT3 inhibitor GPB730 were analyzed by multi-parameter flow cytometry analysis. Since no survival benefit was observed in mice treated with GPB730 alone compared with the vehicle treated group, no further analysis between these groups were performed. Gating strategies for the different immune cell populations are shown in supplementary Fig. 3. The immune cells in RM-1 tumors were analyzed by flow cytometry after 2 weeks of treatment with anti-CTLA-4 or anti-CTLA-4 + GPB730. There was a significant enhanced infiltration of CD45 + cells in tumors of mice treated with anti-CTLA-4 + GPB30 compared to treatment with anti-CTLA-4 alone (Fig. 4a). The frequencies of CD3 + , CD4 + , CD8 + , CD11b + , macrophages or MDSCs among the CD45 + gated cells in tumors were not significantly different between treatment groups (Fig. 4a). No significant correlation between CD45 + and tumor size was observed (Fig. 4b). There were no significant differences in frequencies of CD45 + , CD11b + , macrophages or MDSCs in spleen between treatment groups (supplementary Fig . 4). No differences in NK cell frequency within the tumors was observed between anti-CTLA-4 and the combination anti-CTLA-4 and GPB730 (data not shown).

### GPB730 decreases frequency of tumor Tregs in anti-CTLA-4-treated mice

**Fig. 5:**
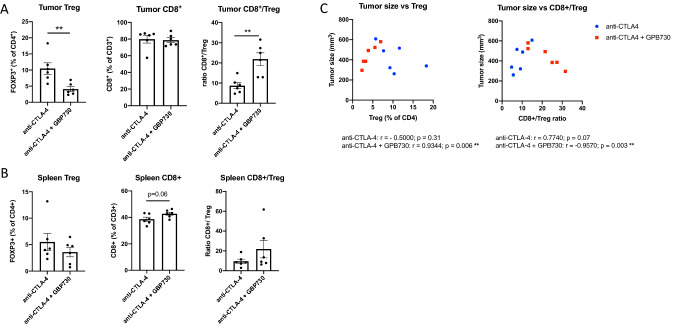
Tumor Tregs are decreased by GPB730 in anti-CTLA-4-treated mice with RM-1 tumors. **a**-**b**. Levels of Tregs (FOXP3 + CD3 + CD4 + gated on CD4 +), CD8 + (gated on CD3 +) and the CD8:Treg ratio in tumors (**a**) and spleens (**b**) of RM-1 tumor bearing mice treated with anti-CTLA-4 (*n* = 6) or anti-CTLA-4 + GPB730 (*n* = 6) for 2 weeks. Data presented as mean ± SEM (**p ≤ 0.001). C. Correlations between tumor Treg levels and tumor size and CD8:Treg ratios and tumor size using Pearson correlation analysis (**p ≤ 0.001)

The levels of Tregs (FOXP3 + CD4 + CD3 +) were significantly decreased in tumors of anti-CTLA-4 + GPB730-treated mice compared to treatment with anti-CTLA-4 alone (Fig. 5a). Furthermore, the CD8 + T cell:Treg ratios were significantly increased by GPB730 in combination with anti-CTLA-4 (Fig. 5a) compared to anti-CTLA-4 alone. Spleen Treg and CD8 + levels did not significantly differ between treatment groups (Fig. 5b). There was a strong and significant correlation between tumor size and Treg tumor levels and tumor size and CD8 + to Treg ratios when combining GPB730 with anti-CTLA-4, which was not observed in anti-CTLA-4-treated mice (Fig. 5c).

Of note, when evaluating a separate cohort comprised of significantly smaller tumors, significantly lower levels of tumor Tregs (1.8 ± 1.2%) were detected in comparison to the Treg levels (7.3 ± 1.3%) in the cohort with larger tumors. We did not detect differences in tumor Treg levels between the treatment groups in the cohort with smaller tumors (data not shown).

### Inflammatory cytokines and chemokines in mouse plasma

**Fig. 6:**
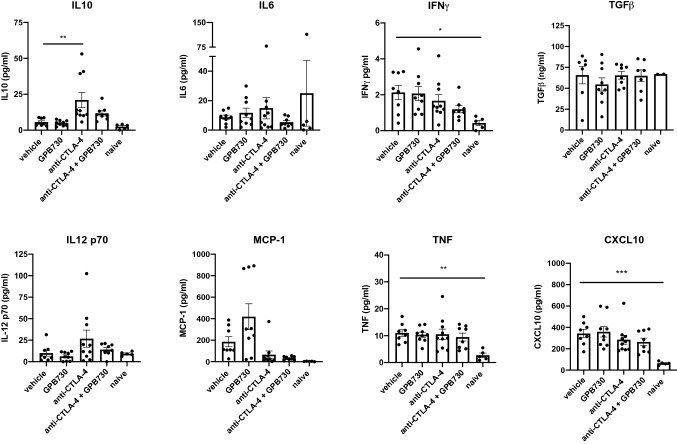
Inflammatory cytokines and chemokines in plasma. Cytokine and chemokine plasma levels in mice with RM-1 tumors treated with vehicle, GPB730, anti-CTLA-4 or anti-CTLA-4 + GPB730 for 2 weeks or in untreated tumor-naive mice without tumors. Data presented as mean ± SEM (vehicle *n* = 8; anti-CTLA-4 n = 10; GPB730 *n* = 10; anti-CTLA-4 + GPB730 *n* = 8; naive *n* = 5). One-way ordinary ANOVA with Dunnetts’s multiple comparisons test in comparison with vehicle group (*p ≤ 0.05; **p ≤ 0.001)

To investigate if antitumor responses are associated with the production of inflammatory soluble factors, the levels of chemokines and inflammatory cytokines (IL10, IL6, IL12p70, TNF, IFNγ, MCP-1, TGFß and CXCL10) were investigated in mouse plasma of RM-1 tumor bearing mice after 2 weeks of treatment with vehicle, GPB730, anti-CTLA-4 or anti-CTLA-4 + GP730 and in tumor naïve mice (Fig. 6). TNF, IFNγ and CXCL10 levels were significantly increased in plasma of mice with RM-1 tumors compared to tumor naïve mice. The IL10 plasma level was significantly increased in mice treated with anti-CTLA-4 compared to vehicle which was attenuated by GPB730 in the combination group. No significant differences in plasma levels of IL6, IL12p70, IFNγ, TNF, TGFβ, MCP-1 or CXCL10 were observed between treatment groups.

## Discussion

We here demonstrate that the STAT3 inhibitor GPB730 enhances the antitumoral effect of anti-CTLA-4 treatment in a syngeneic prostate cancer mouse model.

CTLA-4 blockade by ipilimumab has shown some efficacy in prostate cancer, however with only a subset of patients reaching long-term remission and increased survival [2–4, 9, 18]. Therefore, combination treatments that can enhance the immunotherapeutic effect of anti-CTLA-4 constitute an unmet medical need.

Resistance to immunotherapy involves the immunosuppressive tumor microenvironment which include immunosuppressive immune cells such as MDSCs and Tregs as well as immunosuppressive factors derived from the tumor cells themselves [19, 20]. Targeting an immunosuppressive tumor microenvironment by STAT3 inhibition may consequently enhance the efficacy of immunotherapy [13].

Increased tumor growth after an initial positive response to immunotherapy may be due to acquired resistance mechanisms involving e. g. loss of T cell function, loss of neoantigen and accumulation of immunosuppressive cells [20–22]. Although survival was significantly increased in the combination treatment group with anti-CTLA-4 and GPB730, we did not observe complete tumor regression which was possibly due to acquired resistance. Identification of resistance mechanisms is highly relevant for understanding effective treatment options and regimes.

Intratumoral Tregs are associated with advanced stages and clinical outcome of prostate cancer [23, 24]. Furthermore, the levels of circulating and intratumoral Tregs have been observed to be increased by anti-CTLA-4 treatment in patients with prostate cancer [25, 26]. The presence of immunosuppressive Tregs may hamper antitumoral immunity. This is suggested to be a resistance mechanism to checkpoint inhibitors and targeting Tregs may relieve immunosuppression and enhance the antitumoral effect of immunotherapy [19, 20].

Here, we show that among the different immune cell populations investigated in the current study, only the intratumoral Treg population was significantly decreased when combining GPB730 with anti-CTLA-4 treatment compared to anti-CTLA-4 alone. The enhanced antitumoral activity of the combination treatment may partly be attributed to the increased CD8:Treg ratio in tumors thus enhancing the cytotoxic effect of CD8 + cells. The increased CD8:Treg ratio was inversely correlated to tumor size, further substantiating this hypothesis.

STAT3 is implicated in Treg function [11] and inhibition or ablation of STAT3 has been shown to decrease Treg levels [27, 28]. However, in a study by Kortylewski et al., STAT3 ablation did not decrease the amount of Tregs, but inhibited FOXP3 expression and IL10 production in Tregs thus inhibiting the immunosuppressive capacity [29]. FOXP3 is an essential transcription factor in Tregs regulated by STAT3 and also a co-transcription factor of STAT3 in Tregs [30–32]; the complex enhances the transcription of IL10 among other STAT3 regulated genes. In addition to decreasing the levels of Tregs in anti-CTLA-4-treated tumors, GPB730 may also inhibit the suppressive functions of Tregs, enhancing the cytotoxicity of CD8 + cells; however, functional analysis of Tregs is beyond the scope of the current study.

RM-1 cells are reported to be sensitive to both NK cell cytotoxicity and to T cells [33, 34]. Although NK cells may play an important role in delaying RM-1 tumor progression, these cells probably do not play a role in the enhanced antitumoral effect by GPB730 as no differences in NK cell frequency within the tumors was observed between anti-CTLA-4 and the combination anti-CTLA-4 and GPB730 in this study. A limitation to this study is the lack of functional analysis and immune cell depletion experiments in order to identify the cells directly responsible for antitumoral effects.

Our results are in line with previous findings where a decrease in Tregs are observed by combining anti-CTLA-4 with STAT3 inhibition. The tyrosine kinase inhibitor imatinib, which inhibits STAT3, combined with anti-CTLA-4 decreased intratumoral Treg levels and showed synergistic antitumoral effect [35]. Similar effects were observed when combining the Src inhibitor dasatinib, which also may inhibit STAT3 phosphorylation and activation, with anti-CTLA-4, which lead to increased CD8:Treg ratio in mouse tumors and enhanced the antitumoral effect [36].

STAT3 is mainly activated by phosphorylation of tyrosine 705 (pSTAT3-T705) via various kinases but may also be activated by a non-canonical pathway by phosphorylation of serine 727 (pSTAT3-S727), leading to transcription of distinct sets of genes [11, 37, 38]. In addition, pSTAT3-S727 may exert a non-transcriptional role in mitochondrial activity [37]. pSTAT3-T705 is associated with proliferation and metastasis of tumors besides regulating immunosuppressive factors and function of various immune cells [11, 14, 38]. However, the role of pSTAT3-727 is not as well explored as for pSTAT3-T705. In the context of immune response, pSTAT3-S727 is shown to regulate metabolism and the expression of inflammatory cytokines in macrophages and to regulate transcription of FOXP3 in Tregs [39, 40]. As evident from the IHC and immunofluorescent staining and analysis, pSTAT3-S727 is under these experimental conditions likely to be expressed in cells of the tumor microenvironment (e.g., CD3 + cells) rather than the tumor cells, while pSTAT3-T705 may be expressed in several different cell types in the RM-1 tumors in addition to tumor cells.

We observed an increase in IL10 levels in mouse plasma by anti-CTLA-4 treatment which was attenuated by combining anti-CTLA-4 with GPB730. The observed increase in IL10 levels by anti-CTLA-4 is in accordance with previous studies where serum levels of IL10 were increased by anti-CTLA-4 treatment in malignant mesothelioma [41] and where ipilimumab enhanced the cytokine response, including IL10 and IL6 levels in small-cell lung cancer patients undergoing chemotherapy [42]. IL10 is an immunosuppressive and tumor promoting cytokine expressed by subsets of T cells and myeloid cells e. g. macrophages and dendritic cells among other immune cells [43]. IL10 in Tregs is regulated by STAT3 and lowering levels of IL10 may decrease the immunosuppressive actions of Tregs on cytotoxic T cells [29, 44]. We have previously shown that the STAT3 inhibitor galiellalactone inhibits cytokines such as GM-CSF and IL8 from prostate cancer cells and IL1ß, IL6 and IL10 secretion from monocytes [16].

GPB730 enhanced the CD45 + immune cell infiltration in the prostate cancer mouse tumors of anti-CTLA-4-treated mice which was confirmed by IHC staining of CD45-positive cells in RM-1 tumors. This is in line with observations made in a glioma model where STAT3 inhibition enhanced CD45 + infiltration in tumors [45]. We did however not observe differences in tumor or spleen M-MDSCs or G-MDSCs levels between anti-CTLA-4 and the combination anti-CTLA-4 + GPB730 in this study. Previous studies have shown that anti-CTLA-4 or STAT3 inhibition alone decreases MDSCs in mouse models [28, 36].

In conclusion, STAT3 inhibition by GPB730 enhances the antitumoral activity of anti-CTLA-4 in a prostate cancer mouse model, possibly by blocking STAT3 mediated resistance mechanisms such as Tregs in the immunosuppressive environment. These results raise the possibility that STAT3 inhibition, e.g., by GPB730 in combination with anti-CTLA-4 could constitute a future novel treatment approach in advanced prostate cancer.

## Supplementary Information

Below is the link to the electronic supplementary material.

Supplementary file1 (PDF 57 kb)

Supplementary file2 (PDF 7286 kb)

## Data Availability

Data are available upon reasonable request.
