# Supplementary material for: Inhibition of STAT3 augments antitumor efficacy of anti-CTLA-4 treatment against prostate cancer
Source: Cancer Immunol Immunother. 2021 Mar 31;70(11):3155–66. doi: 10.1007/s00262-021-02915-6 (PMC8505385; doi:10.1007/s00262-021-02915-6)
Supplement: Supplementary file 1 — Supplementary file1 (PDF 57 kb) [file 262_2021_2915_MOESM1_ESM.pdf]

**Supplementary Table 1**. Antibodies used for flow cytometry analysis.

| **Antibody** | **Clone** | **Company** |
|---|---|---|
| anti-CD3-PerCP-Cy5.5 | 145-2C11 | Biolegend |
| Anti-CD3-PE-Cy5 | 145-2C11 | Biolegend |
| anti-CD4-PE-Cy7 | RM4-5 | Biolegend |
| anti-CD11b-PE-Cy7 | M1/70 | Biolegend |
| anti-CD45-BV570 | 30-F11 | Biolegend |
| anti-F4/80-PE-Cy5 | BM8 | Biolegend |
| anti-FOXP3-PE | FJK-165 | eBioscience |
| anti-Ly6C-BV421 | AL-21 | BD Biosciences |
| anti-Ly6G-FITC | 1A8 | Biolegend |
