# Supplementary material for: Inhibition of STAT3 augments antitumor efficacy of anti-CTLA-4 treatment against prostate cancer
Source: Cancer Immunol Immunother. 2021 Mar 31;70(11):3155–66. doi: 10.1007/s00262-021-02915-6 (PMC8505385; doi:10.1007/s00262-021-02915-6)

SUPPLEMENTARY FIGURE CAPTIONS

Supplementary figure 1. Mouse weight. A. Change in mouse weight in survival study. Graph indicates % change in weight from study start to end point for each individual animal (n=11). B. Change in mouse weight in IHC *in vivo* study after 2 weeks of treatment (n= 8-10). Data presented as mean ± SEM.

Supplementary figure 2. Examples of IHC staining patterns in RM-1 tumors. A. CD45 and pSTAT3-T705 immunostainings in RM-1 tumors. Scale bar indicates 1 mm. B. Nuclear and cytoplasmic staining patterns of pSTAT3-S727 in RM-1 tumors. Scale bar indicates 100 µm.

Supplementary figure 3. Flow cytometry gating strategy of dissociated tumors. Representative dot plots of flow cytometry gating strategy of myeloid and lymphoid immune cell populations in dissociated RM-1 tumors.

Supplementary figure 4. Immune cell profile in spleen. Flow cytometry analysis of the immune cell composition in spleen of RM-1 tumor bearing mice treated with anti-CTLA-4 or anti-CTLA-4 + GPB730 for 2 weeks with treatment start 4 days post inoculation. A. CD45+ cells in spleens and proportion of different populations of immune cells among CD45+ gated cells in spleens. B. M-MDSC and G-MDSC in spleen gated on CD11b+ F4/80- cells. Data presented as mean ± SEM (n=6).

Supplementary figure 1.

A.

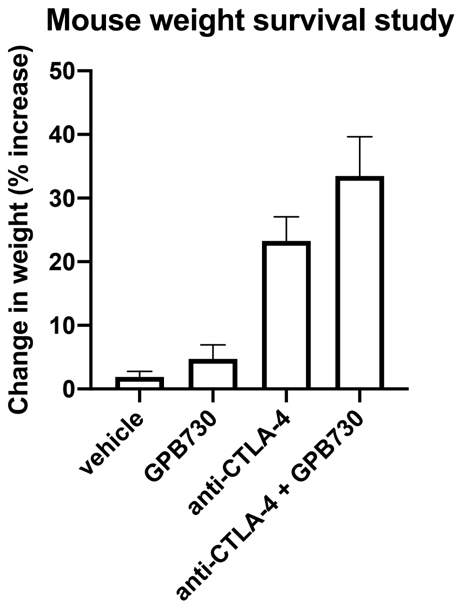

B.

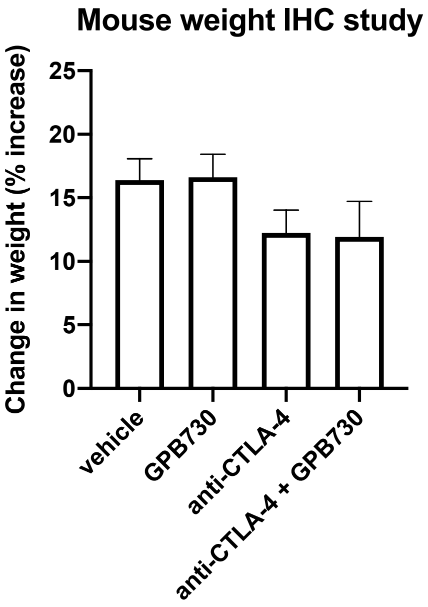

Supplementary figure 2

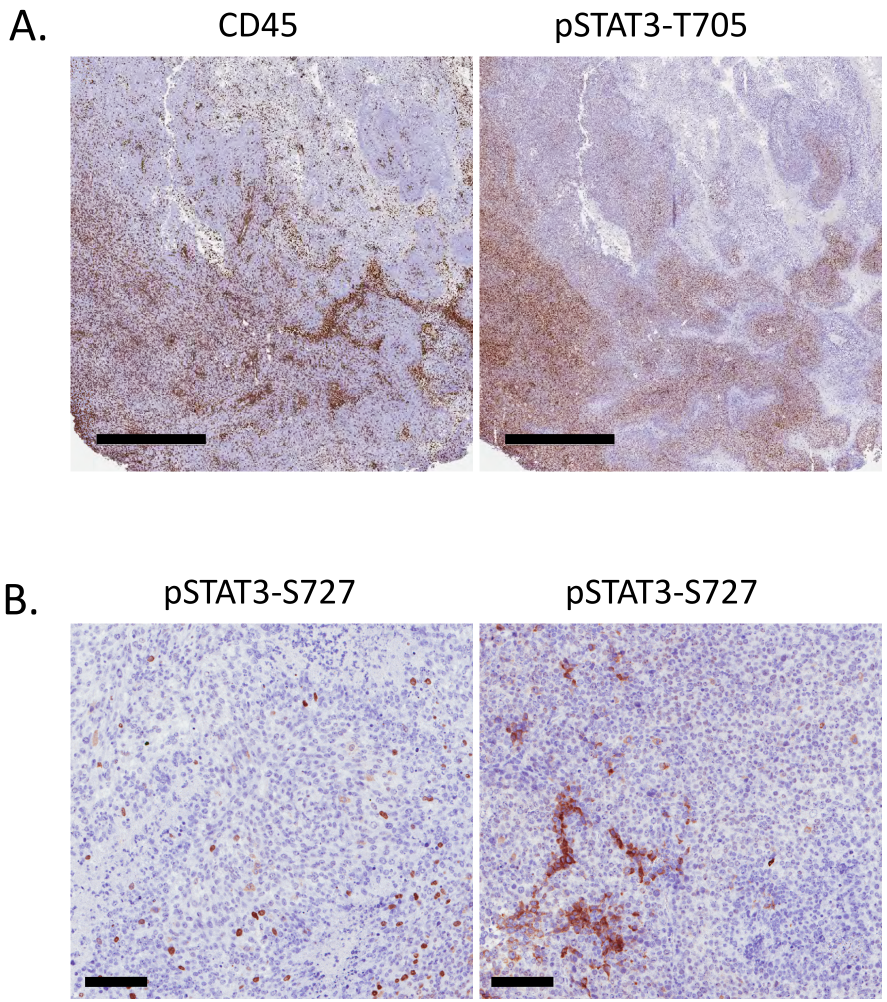

Supplementary figure 3

Gating strategy: myeloid cells (tumor)

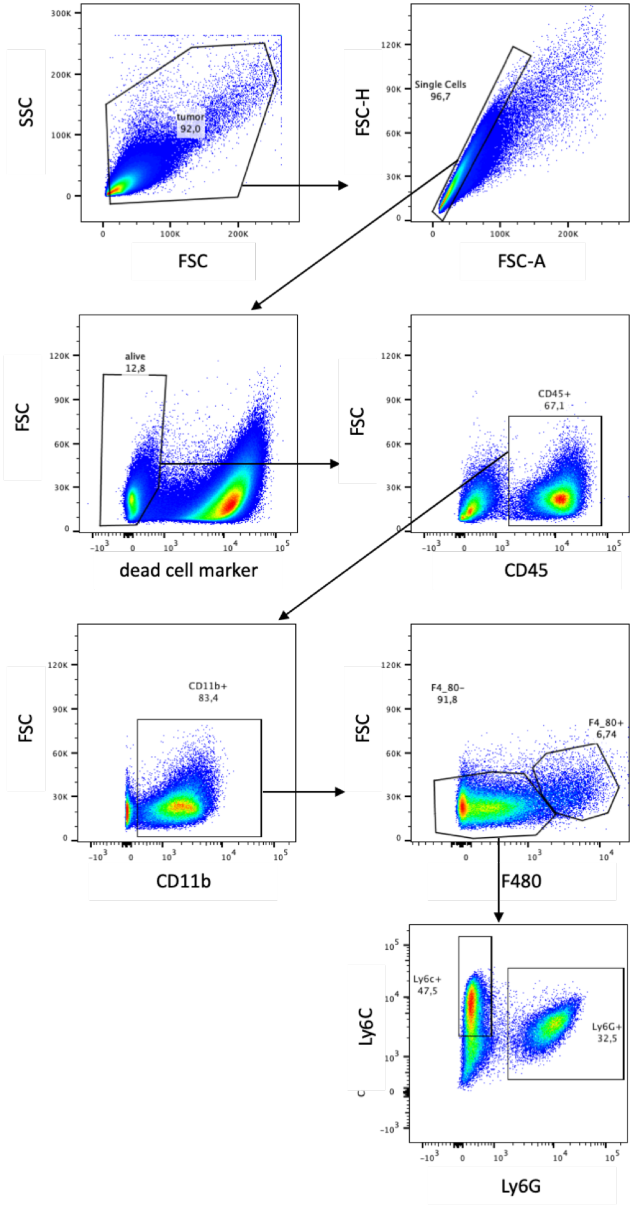

Gating strategy: lymphocytes (tumor)

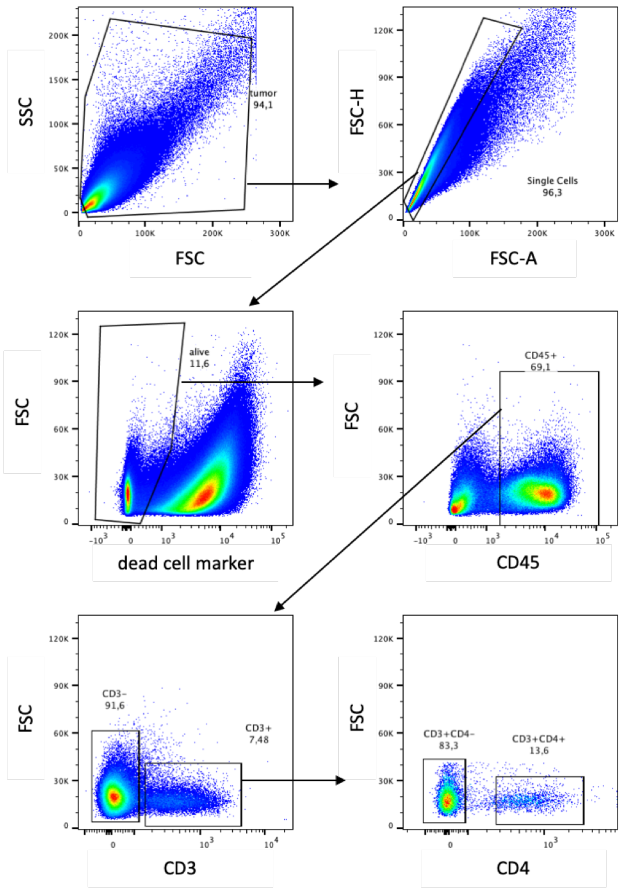

Gating strategy: Tregs (tumor)

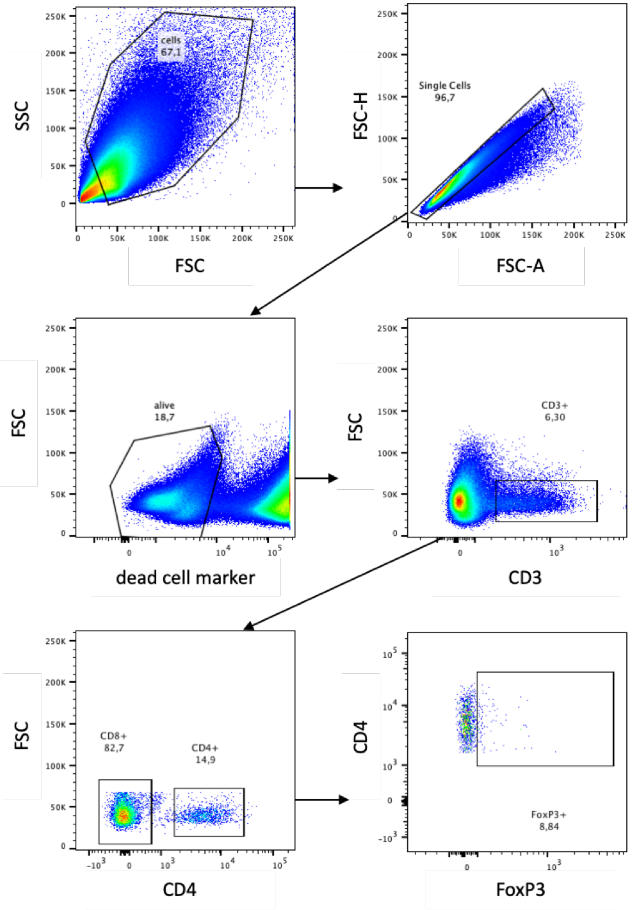

Supplementary figure 4

A.

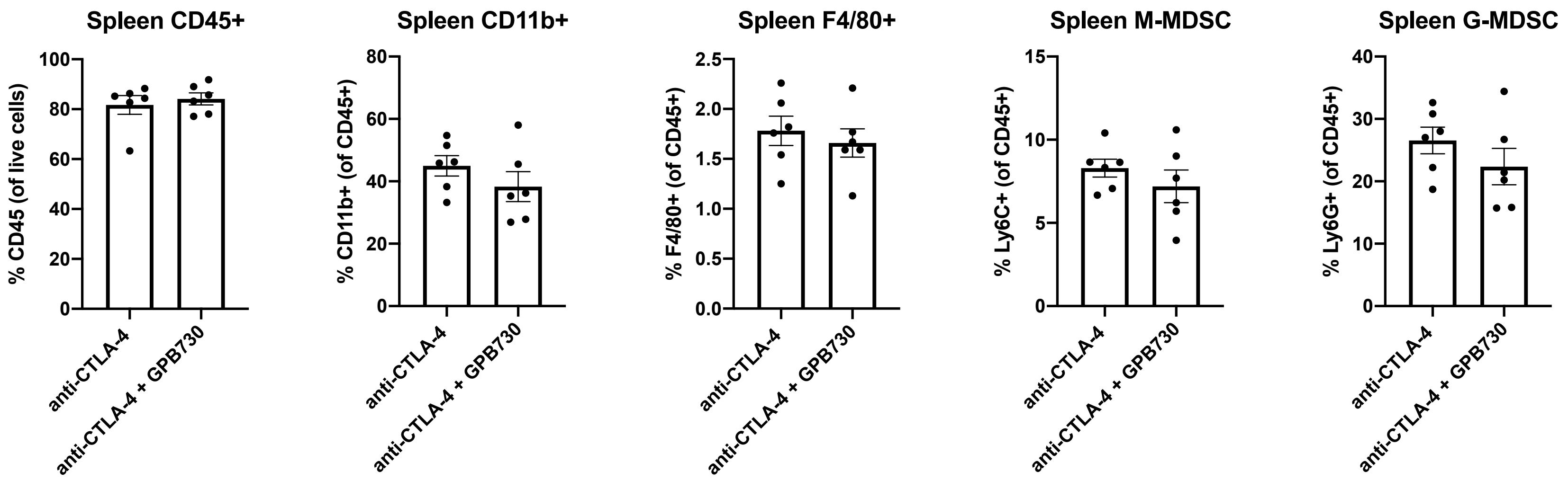

B.

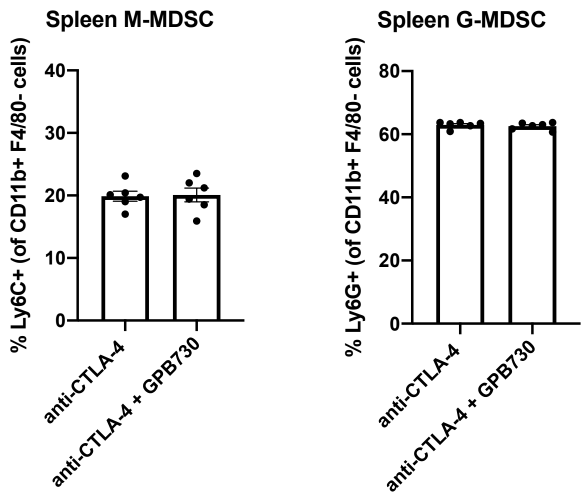

Supplement: Supplementary file 2 — Supplementary file2 (PDF 7286 kb) [file 262_2021_2915_MOESM2_ESM.pdf]
